# Endothelial keratoplasty versus repeat penetrating keratoplasty after failed penetrating keratoplasty: A systematic review and meta-analysis

**DOI:** 10.1371/journal.pone.0180468

**Published:** 2017-07-03

**Authors:** Feng Wang, Tao Zhang, Yan Wei Kang, Jing Liang He, Shi-Ming Li, Shao-Wei Li

**Affiliations:** 1 Department of Ophthalmology, Aier School of Ophthalmology, Central South University, Changsha, Hunan Province, China; 2 Beijing Ophthalmology Visual Science Key Lab, Beijing Institute of Ophthalmology, Capital Medical University, Beijing Tongren Hospital, Beijing, China; University of Illinois at Chicago, UNITED STATES

## Abstract

**Objective:**

This study sought to compare graft survival, graft rejection and the visual acuity outcome of endothelial keratoplasty (EK) with repeat penetrating keratoplasty (PK) after failed PK.

**Methods:**

A systematic literature search with subsequent screening of the identified articles was conducted to obtain potentially eligible randomized clinical trials (RCTs) and comparative cohort studies. To assess the methodological quality of the included studies, the Jadad Scale or Newcastle-Ottawa Scale (NOS) was used based on the study design. To calculate the pooled odds ratios (ORs) for graft survival, graft rejection and the visual acuity outcome with 95% confidential intervals (CIs), a fixed- or random-effects model was applied based on the heterogeneity across studies.

**Results:**

Four comparative cohort studies (n = 649 eyes) comparing the outcome of EK with repeat PK after failed PK were included in this review. These studies were considered high quality, with NOS scores ranging from 6 to 9. The EK group showed a significantly lower risk of graft rejection than the repeat PK group [0.43 (95% CI: 0.23–0.80, *P* = 0.007)]. In addition, no significant differences were observed in a comparison of graft survival and visual acuity (*P* values ranged from 0.81 to 0.97 using the Der-Simonian and Laird random-effects model).

**Conclusions:**

As an alternative to repeat PK, EK after failed PK allows for potential reduction of the risk of graft rejection; however, it does not appear to confer a significant advantage in graft survival or visual acuity.

## Introduction

The diagnosis of graft failure as an indication for keratoplasty has increased in recent years [1]. Patel’s study revealed that between 1989 and 1995, the proportion of regrafts performed at Wills Eye Hospital was 16% (271 of 1,689), compared with 9% (165 of 1,860) during the period from 1983 to 1988 (*P* < 0.01) [2]. Another report showed that regrafting was the most common indication for repeat corneal transplantation between 1990 and 1999, accounting for 40.9% of 784 patients [3]. Because the traditional treatment for failed corneal transplantation is repeat penetrating keratoplasty (PK) surgery, several studies have analyzed the outcomes of repeat PK for graft failure, and comparison of repeat PK with primary PK has revealed that repeat PK can lead to a poorer outcome with lower graft survival than primary PK [4–6].

As an alternative, endothelial keratoplasty (EK) after failed PK has attracted increasing attention [7–13]. In terms of the primary graft, the main advantages of EK over PK include rapid postoperative visual rehabilitation and less allograft rejection [14–19]. With these advantages, EK after a previous failed PK can potentially reduce the risk of rejection compared with repeat PK [20, 21]. Previous studies have found that the graft survival rates of EK after failed PK are approximately 55–100% at 1 year. Recently, several observational studies compared the outcomes of EK after a previously failed PK with repeat PK, although inconsistent results were reported. Ang *et al*. [22] reported that repeat PK was a significant risk factor for graft failure compared with EK after PK with an initial indication of bullous keratopathy [HR: 10.17 (95% confidence interval (CI): 1.10–93.63; *P* = 0.041)], while Keane *et al*. [23] suggested that repeat PK might have a better graft survival outcome than EK after a failed PK that was initially performed for keratoconus or pseudophakic bullous keratopathy. Therefore, we performed this meta-analysis to compare graft survival, graft rejection and the visual outcome of EK with repeat PK after failed PK.

## Materials and methods

### Literature search strategy

We followed the standard literature search guidelines to develop this literature search strategy [24]. The search question and keywords were defined through consultation with a technical expert panel (S1 Appendix). Then, a thorough search strategy was implemented to produce the highest return of relevant clinical studies. The keywords that formed the basis of the search included “failed penetrating keratoplasty”, “endothelial keratoplasty”, “endothelial rejection”, “graft failure” and synonyms. Among these terms, endothelial rejection was defined as the presence of anterior chamber inflammation requiring an unscheduled increase in topical corticosteroid treatment, and graft failure was defined as irreversible loss of corneal clarity resulting from endothelial decompensation in consecutive clinic visits. Literature was obtained from an exhaustive search of the following databases: Cochrane, PubMed, Ovid MEDLINE (January 1946 to January 2017) and Ovid EMBASE (January 1974 to January 2017). After a bibliographic record was obtained, duplicates were removed with EndNote. References from publications that passed the first level of screening were manually searched to acquire further unique resources. The search was initially performed in November 2015, and monthly updates were performed until January 27, 2017.

### Selection criteria

Two authors (FW and TZ) independently screened the titles and abstracts of the studies from the electronic databases to identify all potentially eligible studies. Any uncertainties or discrepancies between the two authors were resolved through consensus after rechecking the source data and consulting with a third party. Then, full articles were read to include the studies that met the following criteria: (1) study design: randomized clinical trial (RCT) or comparative cohort study; (2) population: patients who had undergone either repeat PK or EK for a failed graft; (3) sample size: no fewer than 20 eyes; (4) outcomes: graft survival, graft rejection, and visual acuity; and (5) article language: English. Reviews, letters and conference abstracts that did not provide sufficient information were excluded. We searched the reference lists in the retrieved articles for additional trials and then used PubMed to find studies that cited the identified trials.

### Data extraction and quality assessment

Data were independently extracted by two authors (FW and TZ) using a unified data extraction form. Any disagreement was resolved by rechecking and discussion. For each included study, detailed information on the title, surname of the first author, publication year, country, study design, sample size, characteristics of the patients, time of follow-up, Kaplan-Meier survival analysis, and number of graft rejection cases was extracted. The methodological quality of the included studies was examined using the Jadad Scale [25] for RCTs and the Newcastle-Ottawa Scale (NOS) [26] for cohort studies, considering the following domains: selection of study groups, comparability of study groups and assessment of outcome. The Jadad Scale ranges from 0 to 5, while the NOS ranges from 0 to 9. If the Jadad score was ≥ 3 points, the trial was considered a high-quality study, while if the Jadad score was ≤ 2 points, the study was considered low quality. In terms of cohort studies, NOS scores of 0–3, 4–6, and 7–9 were considered to indicate low, moderate and high quality, respectively.

### Statistical analysis

In cases when the data were sufficient, meta-analysis was performed based on the defined outcomes for the comparisons of EK procedures with repeat PK procedures. Pooled odds ratios (ORs) were calculated to compare graft survival, graft rejection and the visual acuity outcome between the two groups. Zero total event data were also generated using the constant correction method, in which 0.5 was added to each cell of the 2×2 table for any such study using RevMan software. The heterogeneity across studies was assessed using the Cochrane Q test and *I*^*2*^ statistic. A fixed-effect model was used to calculate estimates unless there was significant heterogeneity (*P* > 0.1 and *I*^2^ < 50%), in which case a random-effects model was used. In the case of significant heterogeneity in the data combination, a sensitivity analysis was conducted to provide some possible explanation or insight. Publication bias was assessed if the number of involved investigations was ≥ 10 [27]. All statistical calculations were performed using Review Manager 5.3 software. A *P*-value < 0.05 was considered statistically significant.

## Results

### Literature search

**Fig 1:**
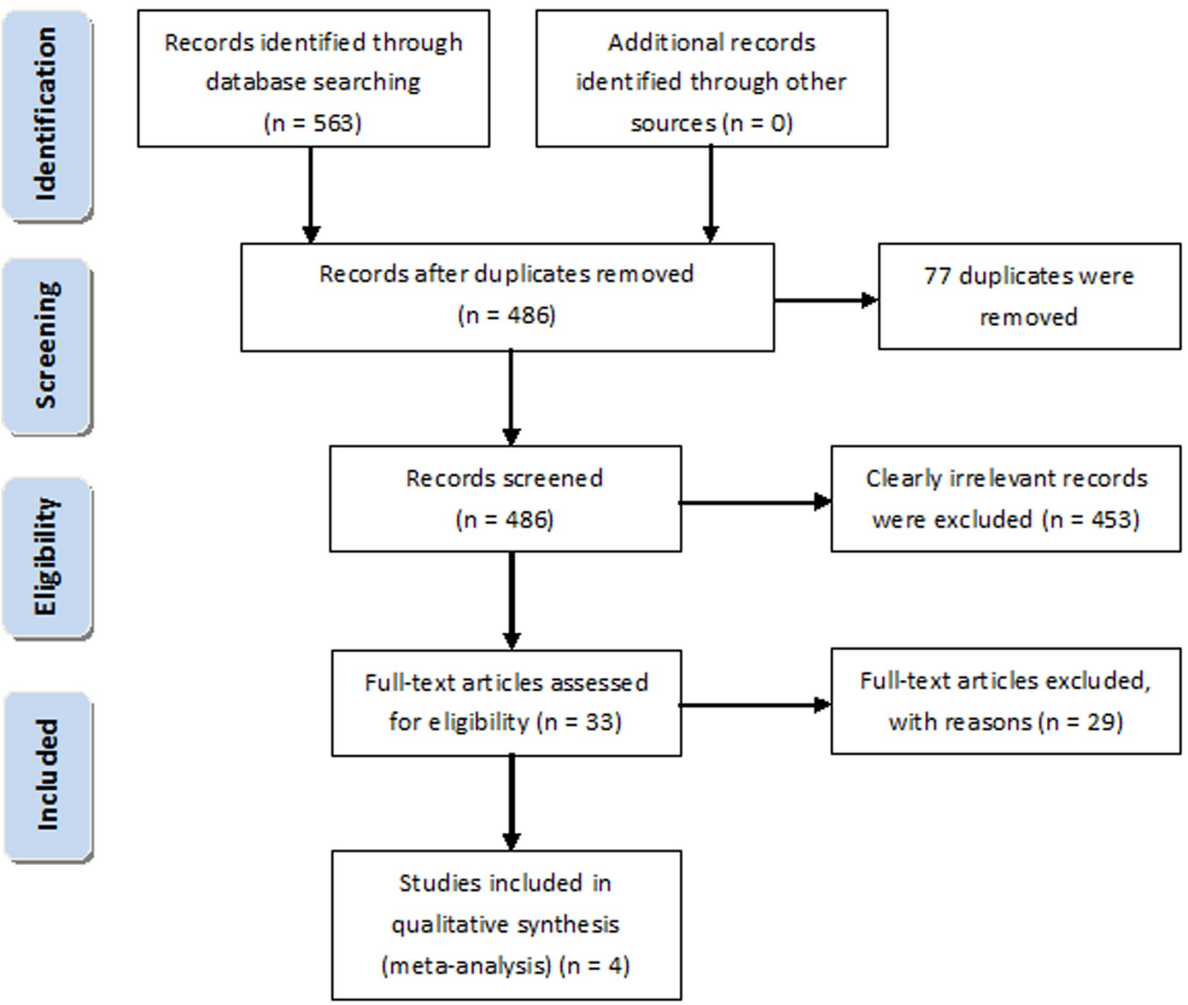
Flow chart of publication selection.

The study selection process is shown in Fig 1. A total of 563 papers were identified by electronic search. After 77 duplicates were removed, the titles and abstracts of the remaining 486 articles were screened. A total of 453 articles were excluded because they were irrelevant. After reading and evaluating the remaining 33 publications, 29 were excluded because they either did not have a long-term follow-up period, did not have a sufficiently large sample size of participants or did not provide the desired outcomes (S2 Appendix). Finally, four cohort studies were included in the present meta-analysis. All cohort studies included patients who underwent repeat PK or EK following failed PK. The definitions of “graft failure” and “graft rejection” were consistent across studies.

### Study characteristics

We did not identify any RCTs comparing EK with repeat PK for patients after a prior failed PK. Nonetheless, four comparative cohort studies performed comparisons and presented a report of the outcomes for graft rejection, graft survival and visual acuity. The primary characteristics of the included studies are summarized in Table 1.

**Table 1:** Characteristics of the studies included in the meta-analysis.

Author and year of publication	Country	Study design	Number of eyes	Patients				Maximum follow-up years	Quality score
				EK		Repeat PK			
				Number	Mean age (years)	Number	Mean age (years)		
**Kitzmann *et al*. 2012**^**[**^^**28**^^**]**^	USA	Retrospective cohort	24	7	81	17	71	3.0	6
**Ang *et al*. 2014**^**[**^^**22**^^**]**^	Singapore	Retrospective cohort	113	32	64.4	81	70.9	5.0	9
**Ramamurthy *et al*. 2016**^**[**^^**29**^^**]**^	India	Retrospective cohort	112	45	39	67	40	5.0	9
**Keane *et al*. 2016**^**[**^^**23**^^**]**^	Australia	Prospective cohort	400	65	\	335	\	6.8	8

### Study quality

The NOS was used to evaluate the quality of the included studies (S3 Appendix). All the studies were of high quality, with scores ranging from 7 to 9 (Table 1). Among them, three comparative cohort studies were considered high-quality studies (NOS score > 7), while the remaining study (Kitzmann *et al*. 2012 [28]) was considered a moderate-quality study (4 ≤ NOS score ≤ 6).

### Graft survival

**Fig 2:**
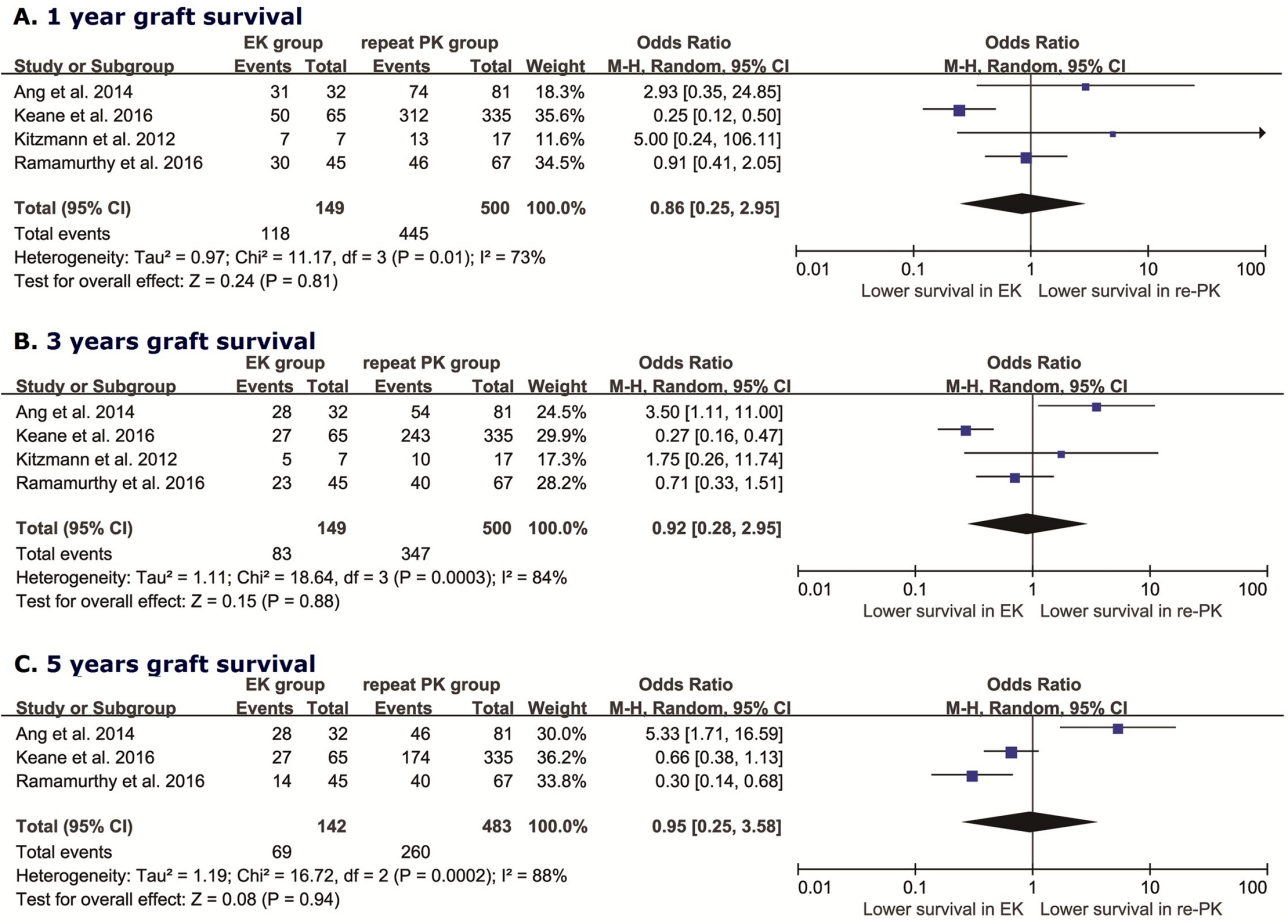
Forest plots of the pooled ORs for the survival of EK versus repeat PK after failed PK. 1 year (**A**); 3 years (**B**), and 5 years (**C**).

Graft survival (1 year, 3 years, and 5 years respectively) of following EK vs. repeat PK after failed PK was compared using the random-effects model, as described by Der-Simonian and Laird, considering both within-study and between-study variability to calculate the summary OR, estimates and corresponding 95% CI. The pooled OR for the rate of graft survival (1 year) in the comparison of EK with repeat PK was 0.86 (95% CI: 0.25–2.95, *I*^*2*^ = 73%, *P*_*heterogeneity*_ = 0.01), as shown in Fig 2(A), while the pooled OR was 0.92 (95% CI: 0.28–2.95, *I*^*2*^ = 84%, *P*_*heterogeneity*_ < 0.001) for 3-year graft survival (Fig 2B). Calculation of the pooled OR for 5-year graft survival was performed by removing the Kitzmann study, as it had a shorter follow-up period, resulting in a value of 0.95 (95% CI: 0.25–3.58, *I*^*2*^ = 88%, *P*_*heterogeneity*_ < 0.001, Fig 2C). There was significant heterogeneity across the included studies, with *I*^*2*^ values ranging from 73 to 88% in pooling of the ORs for graft survival. Considering that meta-regression could not be performed owing to the small number of included studies (n < 10) [30–32], sensitivity analysis with the sequential omission of individual studies was conducted to identify the main source of heterogeneity. However, this approach did not alter the significance of heterogeneity, which ranged from 59 to 94%.

### Graft rejection

**Fig 3:**
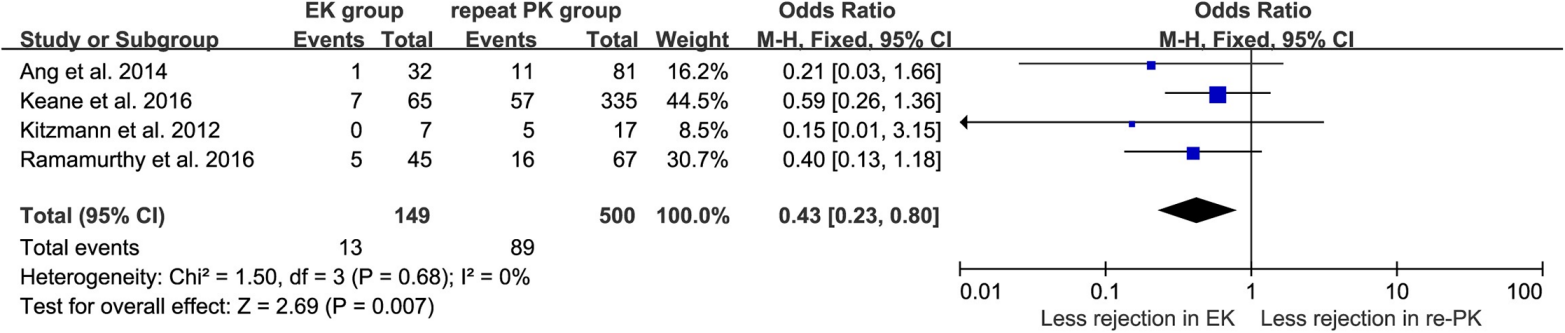
Forest plots of the pooled ORs for graft rejection comparing EK with repeat PK after failed PK.

The estimated overall OR for graft rejection in the comparison of EK with repeat PK was 0.43 (95% CI: 0.23–0.80, *P* = 0.007) using the fixed-effects model, and no heterogeneity was found across studies (*I*^*2*^ = 0%, *P*_*heterogeneity*_ = 0.68, Fig 3). The graft rejection rate of the EK group was significantly lower than that of the repeat PK group. Notably, zero total events existed in the comparison of 1-year graft survival, as well as that of graft rejection, in Kitzmann’s study. The corresponding pooled ORs were calculated with inclusion of the zero total event data using the constant correction method, which produced the results mentioned above. Similar to Friedrich’s study [33], our additional calculations of the pooled ORs excluding the zero total event data did not significantly change the results (S4 Appendix).

### Visual acuity

**Fig 4:**
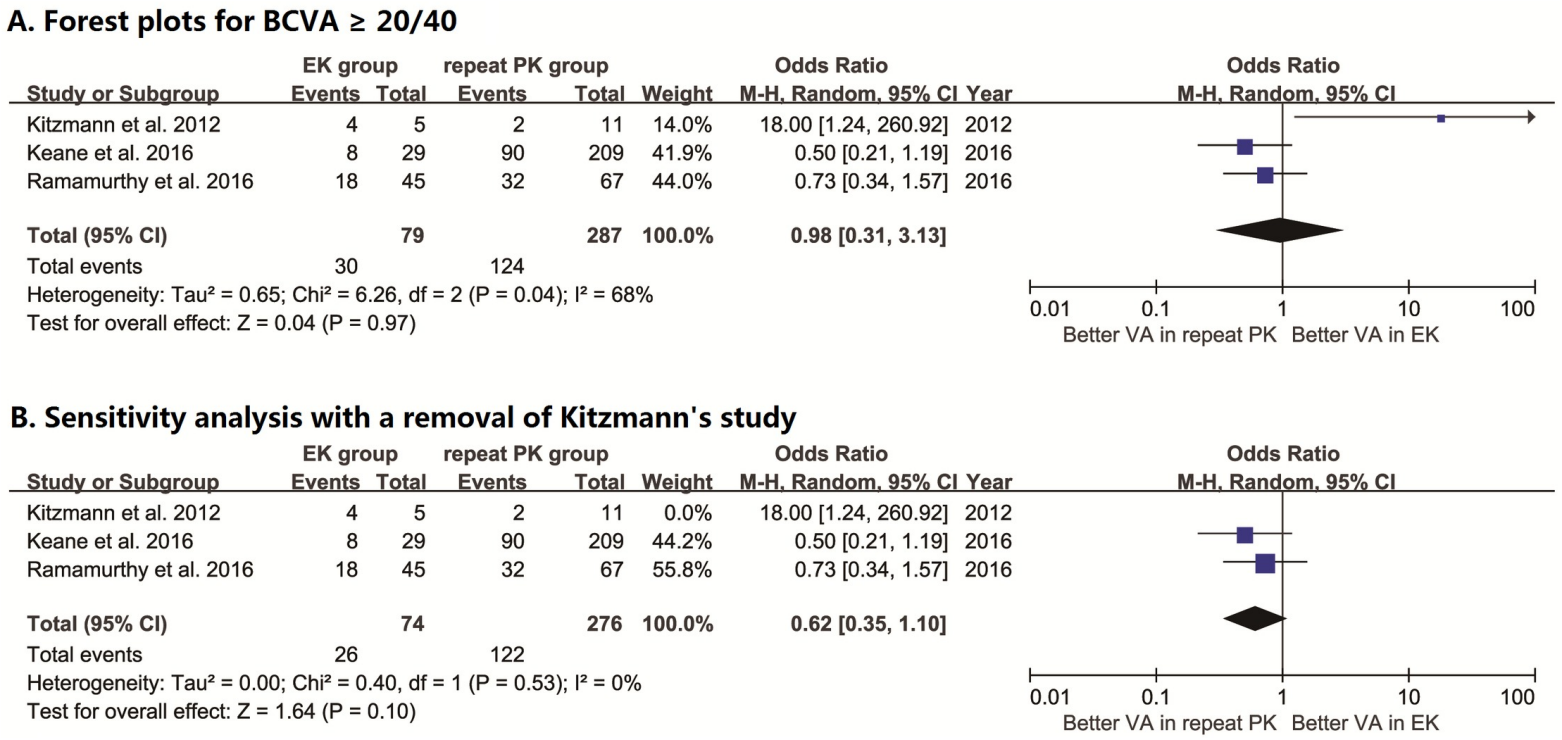
Forest plots for visual outcome. (**A**) Pooled OR for the proportion of grafts that regained a BCVA ≥ 20/40 comparing EK with repeat PK after failed PK; (**B**) sensitivity analysis with removal of Kitzmann’s study.

Few detailed data on the improvement of visual acuity were reported in the four included studies. Three reported the number (or proportion) of grafts that regained a best-corrected visual acuity (BCVA) ≥ 20/40 in the final follow-up, and only two studies reported the mean visual acuity. To quantify the visual outcome for comparison, the pooled OR for the proportion of grafts that regained a BCVA ≥ 20/40 was calculated. The random-effects model was used, which yielded a value of 0.98 (95% CI: 0.31–3.13, *I*^*2*^ = 68%, *P*_*heterogeneity*_ = 0.04), as shown in Fig 4(A). There was significant heterogeneity among the included studies. The results of sensitivity analysis performed with sequential omission of individual studies revealed that Kitzmann’s study was the main source of the heterogeneity, but the corresponding adjusted combined effect size was not significant (*P* = 0.10, Fig 4B).

## Discussion

In our meta-analysis of four cohort studies, EK showed a lower graft survival rate than repeat PK after failed PK, although there was significant heterogeneity among the included studies. Although sensitivity analysis did not help to identify the source of heterogeneity among the included studies, there was significant bias in the selection of patients, which may represent one source of heterogeneity. In Ramamurthy’s study [29], the included patients were specifically confined to those with an indication of a failed therapeutic graft, a high-risk indication for EK surgery. In Keane’s study, all eyes in the EK group exhibited a history of endothelial failure or rejection (S5 Appendix), which was found to have a significant influence on graft survival, while many eyes in the repeat PK group exhibited no history of endothelial failure or rejection. Although both studies had a relatively large sample size, corresponding subgroup analysis of the original PK preoperative or postoperative factors could not be performed because of the lack of relevant data. Additionally, Kitzmann’s study reported no significant difference in graft survival of the two groups with a smaller sample size, while Ang’s retrospective study reported significantly better graft survival of the EK group versus the repeat PK after failed PK group in 113 eyes. Heterogeneity may also have been present because the original clinical data were obtained from multiple centers with varying surgical techniques and surgeon experience levels; in addition, these studies compared a relatively newer EK procedure with established PK techniques.

Consistent with the evidence from previous studies [6, 7, 12, 34], our meta-analysis revealed that subsequent EK led to a significantly lower risk of graft rejection than repeat PK after failed PK. The advantage of performing EK over PK is the potential for a reduced risk of graft rejection; this is also consistent with evidence from studies showing a lower risk of endothelial rejection in primary EK compared to primary PK [11, 35, 36].

In terms of the visual acuity outcomes, the pooled OR for the proportion of grafts that regained a BCVA ≥ 20/40 showed a value of 0.75 with significant heterogeneity. Although the sensitivity analysis was performed to identify the source of heterogeneity, the analysis of the comparison of visual outcomes was not statistically significant. Among the included studies, Ang’s study reported significant improvement in the postoperative BCVA in both groups, although there were no significant differences in BCVA at the 1-year follow-up between the two groups. Additionally, Kitzmann’s and Ramamurthy’s studies produced the same results, while Keane’s study did not perform a comparison because of the small number of survival grafts in the EK group. Nonetheless, most case series have reported significant improvement in BCVA after EK for failed PK [10, 37, 38]. Anshu’s study investigated 60 eyes and found a significant improvement in the median BCVA from the preoperative 1.00 logMAR to the 0.40 logMAR at 1 year (*P* < 0.0001) [39]. Heitor de Paula’s study reported an improvement in the BCVA from 1.43 logMAR preoperatively to 0.55 logMAR at 1 year in 22 eyes (*P* = 0.001) [9]. For repeat PKs, Patel *et al*. [2] reported that 111 of 150 repeat grafts (74%) remained clear, with a mean follow-up of 3.9 years, and that only 30% of repeat grafts achieved 20/40 vision. Similarly, Al-Mezaine *et al*. [34] reported that 114 of 210 repeat PKs (54%) remained clear during an average follow-up period of 43 months and that 4.8% of repeat PKPs showed 20/40 vision at the last follow-up visit. Although increasing studies have indicated that EK can result in effective and rapid visual recovery, further comparative studies with a larger sample size and longer follow-up are required.

## Conclusion

In conclusion, our present study found that EK led to a significantly lower risk of graft rejection than repeat PK after failed PK, and this result is consistent with the evidence from previous studies. However, no significant differences in the graft survival rate or visual outcome were observed between the two groups. Although these results were limited and inconclusive because of the small number of comparative cohort studies and the selection bias of the included studies, the findings indicate that EK is a better alternative to repeat PK for second corneal transplantation because of its lower graft rejection rate, especially for patients whose prior graft failure was a result of endothelial edema or endothelial rejection. Nevertheless, further comparative studies with a larger sample size, longer follow-up period, identical preoperative factors, and well-described visual acuity outcome measurements are needed to increase understanding of the benefits of EK versus repeat PK in treating failed PK.

## Supporting information

S1 Appendix Electronic search strategy. (DOCX)

S2 Appendix Reasons for exclusion. (DOCX)

S3 Appendix NOS quality of the included studies. (DOCX)

S4 Appendix Zero total event data. (PDF)

S5 Appendix Noteworthy reasons for original PK failure. (DOCX)

S1 File PRISMA checklist. (DOC)
