# Supplementary material for: Endothelial keratoplasty versus repeat penetrating keratoplasty after failed penetrating keratoplasty: A systematic review and meta-analysis
Source: PLoS One. 2017 Jul 3;12(7):e0180468. doi: 10.1371/journal.pone.0180468 (PMC5495398; doi:10.1371/journal.pone.0180468)
Supplement: S1 Appendix — (DOCX) [file pone.0180468.s001.docx]

**S1 Appendix**

**Electronic search strategy**

**Cochrane:**

#1 failed penetrating keratoplasty

#2 fail* near/3 penetrat* near/3 keratoplast*

#3 fail* near/3 PK*

#4 #1 or #2 or #3

#5 MeSH descriptor: Keratoplasty, Penetrating

#6 penetrat* near/3 keratoplast*

#7 PKP

#8 endothel* near/3 keratoplast*

#9 MeSH descriptor: Desemet Membrane

#10 descemet* near/6 keratoplast*

#11 DLEK or DSEK or DMEK or DSAEK

#12 #5 or #6 or #7 or #8 or #9 or #10 or #11

#13 #4 and #12

**PubMed:**

#1 failed penetrating keratoplasty/

#2 (fail$ adj3 penetrat$ adj3 keratoplast$).tw.

#3 (fail$ adj3 PK$).tw.

#4 #1 or #2 or #3

#5 MeSH descriptor: Keratoplasty, Penetrating

#6 penetrat$ adj3 keratoplast$

#7 PKP

#8 endothel* near/3 keratoplast*

#9 MeSH descriptor: Desemet Membrane

#10 descemet* near/6 keratoplast*

#11 DLEK or DSEK or DMEK or DSAEK

#12 #5 or #6 or #7 or #8 or #9 or #10 or #11

#13 #4 and #12

**Ovid MEDLINE (January 1946 to January 2017):**

#1 fail* penetrating keratoplasty OR fail* PKP OR failed therapeutic keratoplasty

#2 penetrating keratoplasty OR PKP OR endothelial keratoplasty OR EK OR DLEK or DSEK or DMEK or DSAEK

#3 #1 AND #2

**Ovid EMBASE (January 1974 to January 2017):**

#1 fail* penetrating keratoplasty OR fail* PKP OR failed therapeutic keratoplasty

#2 penetrating keratoplasty OR PKP OR endothelial keratoplasty OR EK OR DLEK or DSEK or DMEK or DSAEK

#3 #1 AND #2
