# Supplementary material for: Endothelial keratoplasty versus repeat penetrating keratoplasty after failed penetrating keratoplasty: A systematic review and meta-analysis
Source: PLoS One. 2017 Jul 3;12(7):e0180468. doi: 10.1371/journal.pone.0180468 (PMC5495398; doi:10.1371/journal.pone.0180468)
Supplement: S2 Appendix — (DOCX) [file pone.0180468.s002.docx]

**S2 Appendix**

**Table S1. Excluded studies with reasons.**

| **Study** | **First Author** | **Year** | **Reason for exclusion** |
| --- | --- | --- | --- |
| **1** | Anthony J. Aldave | 2000 | Non-comparative study |
| **2** | Arundhati Anshu | 2013 | Non-comparative study |
| **3** | Arundhati Anshu | 2011 | Non-comparative study |
| **4** | Tobias Brockmann | 2015 | Non-comparative study |
| **5** | Sunita Chaurasia | 2014 | Non-comparative study |
| **6** | Margareta Claesson | 2013 | Comparison is between regrafts with first grafts rather than EK with repeat PK after failed PK. |
| **7** | John L. Clements | 2011 | Non-comparative study |
| **8** | Douglas J. Covert | 2007 | Non-comparative study |
| **9** | Sybille Graef | 2011 | Cases report, non-comparative study |
| **10** | Enken Gundlach | 2015 | Small sample size, non-comparative study |
| **11** | Ken Hayashi | 2013 | Non-comparative study |
| **12** | Fernando Heitor de Paula | 2012 | Non-comparative study |
| **13** | Anisha A. Jangi | 2012 | Non-comparative study |
| **14** | Thu-Lan Kelly | 2011 | Non-comparative study |
| **15** | Peter Kim | 2011 | Cases report, non-comparative study |
| **16** | George D Kymionis | 2013 | Case report, non-comparative study |
| **17** | Bryan S Lee | 2011 | Non-comparative study |
| **18** | Joseph J. K. Ma | 2005 | Comparison is between repeat PK with Boston Keratoprosthesis rather than EK. |
| **19** | Mark D. Mifflin | 2011 | A letter rather than a cohort study |
| **20** | Danny Mitry | 2014 | A multicenter retrospective interventional case series rather than a comparative study |
| **21** | Jennifer M Nottage | 2012 | A retrospective study rather than a comparative study |
| **22** | Iben B. Pedersen | 2015 | The included patients underwent PK or EK not for the treatment of failed penetrating keratoplasty. |
| **23** | Francis W. Price | 2011 | Non-comparative study |
| **24** | Sloan W. Rush | 2011 | A retrospective case series, non-comparative study |
| **25** | Marianne O. Price | 2011 | Meeting abstract, non-comparative study |
| **26** | Michael D. Straiko | 2011 | A Retrospective study, non-comparative study, small sample size |
| **27** | Janine N. Tarantino-Scherrer | 2015 | Non-comparative study |
| **28** | Daniel J. Weisbrod | 2003 | Comparison is between repeat PK with primary PK rather than EK after a failed PK. |
| **29** | K. Zitte | 2015 | German language, non-comparative study |
