# Supplementary material for: Endothelial keratoplasty versus repeat penetrating keratoplasty after failed penetrating keratoplasty: A systematic review and meta-analysis
Source: PLoS One. 2017 Jul 3;12(7):e0180468. doi: 10.1371/journal.pone.0180468 (PMC5495398; doi:10.1371/journal.pone.0180468)
Supplement: S3 Appendix — (DOCX) [file pone.0180468.s003.docx]

**S3 Appendix**

**Table S2. Study quality in the including investigations using Newcastle-Ottawa-Scale (NOS)**

| **Study** | **Representativeness of the exposed cohort** | **Selection of the non-exposed cohort** | **Ascertainment of exposure** | **Demonstration that outcome of interest was not present at start of study** | **Comparability of cohorts on the basis of the design^a^ or analysis** | **Assessment of outcome** | **Was follow-up long enough for outcomes to occur** | **Adequacy of follow-up of cohorts** | **Quality score** |
| --- | --- | --- | --- | --- | --- | --- | --- | --- | --- |
| **Kitzmann, 2012** | ☆ | ★ | ★ | ★ | ★★ | ☆ | ☆ | ★ | 6 |
| **Ang, 2014** | ★ | ★ | ★ | ★ | ★★ | ★ | ★ | ★ | 9 |
| **Ramamurthy, 2016** | ★ | ★ | ★ | ★ | ★★ | ★ | ★ | ★ | 9 |
| **Keane, 2016** | ★ | ★ | ★ | ★ | ★ | ★ | ★ | ★ | 8 |

*^a^* A maximum of 2 stars can be allotted in this category, one for characteristics of patients, the other for other controlled factors.
