# Supplementary material for: Endothelial keratoplasty versus repeat penetrating keratoplasty after failed penetrating keratoplasty: A systematic review and meta-analysis
Source: PLoS One. 2017 Jul 3;12(7):e0180468. doi: 10.1371/journal.pone.0180468 (PMC5495398; doi:10.1371/journal.pone.0180468)
Supplement: S4 Appendix — (PDF) [file pone.0180468.s004.pdf]

S4 Appendix

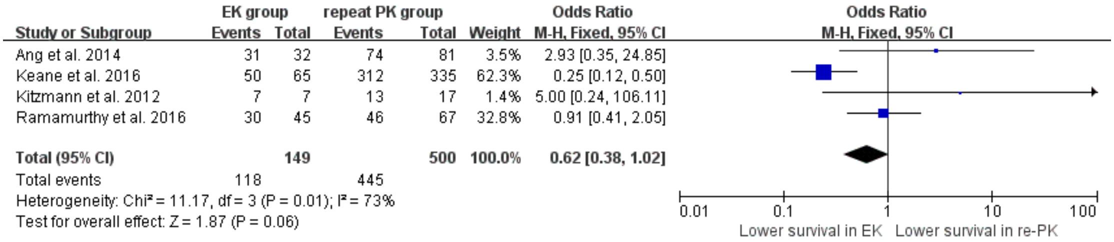

Study or Subgroup	EK group Events	EK group Total	repeat PK group Events	repeat PK group Total	Weight	Odds Ratio M-H, Fixed, 95% CI
Ang et al. 2014	31	32	74	81	3.5%	2.93 [0.35, 24.85]
Keane et al. 2016	50	65	312	335	62.3%	0.25 [0.12, 0.50]
Kitzmann et al. 2012	7	7	13	17	1.4%	5.00 [0.24, 106.11]
Ramamurthy et al. 2016	30	45	46	67	32.8%	0.91 [0.41, 2.05]
**Total (95% CI)**		**149**		**500**	**100.0%**	**0.62 [0.38, 1.02]**
Total events	118		445			

Heterogeneity: Chi² = 11.17, df = 3 (P = 0.01); I² = 73%
Test for overall effect: Z = 1.87 (P = 0.06)

Fig S1. Forest plot including zero total event in 1-year survival

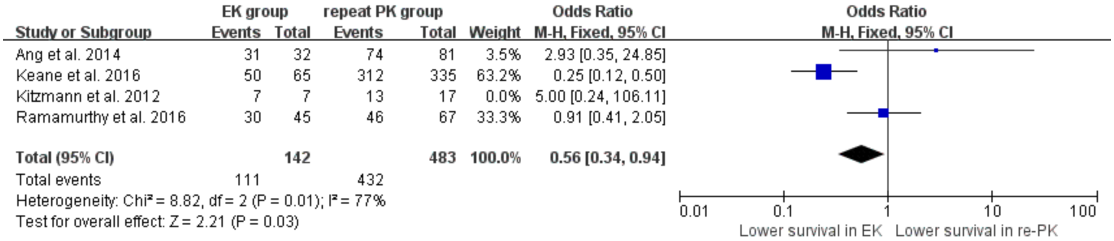

Study or Subgroup	EK group Events	EK group Total	repeat PK group Events	repeat PK group Total	Weight	Odds Ratio M-H, Fixed, 95% CI
Ang et al. 2014	31	32	74	81	3.5%	2.93 [0.35, 24.85]
Keane et al. 2016	50	65	312	335	63.2%	0.25 [0.12, 0.50]
Kitzmann et al. 2012	7	7	13	17	0.0%	5.00 [0.24, 106.11]
Ramamurthy et al. 2016	30	45	46	67	33.3%	0.91 [0.41, 2.05]
**Total (95% CI)**		**142**		**483**	**100.0%**	**0.56 [0.34, 0.94]**
Total events	111		432			

Heterogeneity: Chi² = 8.82, df = 2 (P = 0.01); I² = 77%
Test for overall effect: Z = 2.21 (P = 0.03)

Fig S2. Forest plot excluding zero total event in 1-year survival

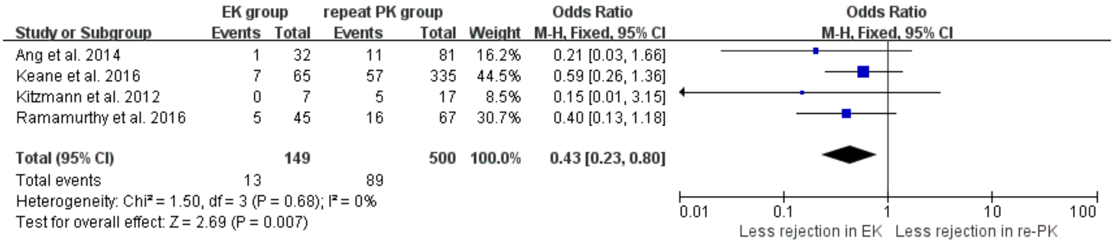

Study or Subgroup	EK group Events	EK group Total	repeat PK group Events	repeat PK group Total	Weight	Odds Ratio M-H, Fixed, 95% CI
Ang et al. 2014	1	32	11	81	16.2%	0.21 [0.03, 1.66]
Keane et al. 2016	7	65	57	335	44.5%	0.59 [0.26, 1.36]
Kitzmann et al. 2012	0	7	5	17	8.5%	0.15 [0.01, 3.15]
Ramamurthy et al. 2016	5	45	16	67	30.7%	0.40 [0.13, 1.18]
**Total (95% CI)**		**149**		**500**	**100.0%**	**0.43 [0.23, 0.80]**
Total events	13		89			

Heterogeneity: Chi² = 1.50, df = 3 (P = 0.68); I² = 0%
Test for overall effect: Z = 2.69 (P = 0.007)

Fig S3. Forest plot including zero total event in graft rejection

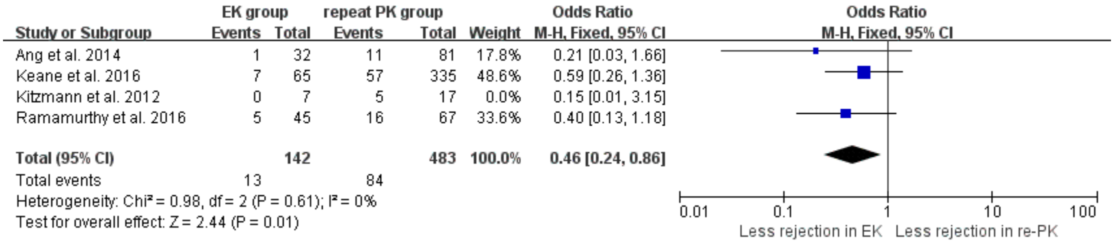

Study or Subgroup	EK group Events	EK group Total	repeat PK group Events	repeat PK group Total	Weight	Odds Ratio M-H, Fixed, 95% CI
Ang et al. 2014	1	32	11	81	17.8%	0.21 [0.03, 1.66]
Keane et al. 2016	7	65	57	335	48.6%	0.59 [0.26, 1.36]
Kitzmann et al. 2012	0	7	5	17	0.0%	0.15 [0.01, 3.15]
Ramamurthy et al. 2016	5	45	16	67	33.6%	0.40 [0.13, 1.18]
**Total (95% CI)**		**142**		**483**	**100.0%**	**0.46 [0.24, 0.86]**
Total events	13		84			

Heterogeneity: Chi² = 0.98, df = 2 (P = 0.61); I² = 0%
Test for overall effect: Z = 2.44 (P = 0.01)

Fig S4. Forest plot excluding zero total event in graft rejection
