# Supplementary material for: Endothelial keratoplasty versus repeat penetrating keratoplasty after failed penetrating keratoplasty: A systematic review and meta-analysis
Source: PLoS One. 2017 Jul 3;12(7):e0180468. doi: 10.1371/journal.pone.0180468 (PMC5495398; doi:10.1371/journal.pone.0180468)
Supplement: S5 Appendix — (DOCX) [file pone.0180468.s005.docx]

**S5 Appendix**

**Table S3. Noteworthy reasons for original PK failure.**

| **Study** | **Reason for original PK failure** | **Percentage of corneal grafts** | |
| --- | --- | --- | --- |
|  |  | **PK-PK** | **PK-EK** |
| **Kitzmann 2012** | Endothelial failure | \ | \ |
|  | Graft rejection | \ | \ |
| **Ang 2014** | Endothelial failure | 55.6 % | 78.1 % |
|  | Graft rejection | 27.2 % | 15.6 % |
| **Ramamurthy 2016** | Endothelial failure | \ | \ |
|  | Graft rejection | \ | \ |
| **Keane 2016** | Endothelial failure | 32.2 % | 69.2 % |
|  | Graft rejection | 23.3 % | 30.8 % |
