# Supplementary material for: Endothelial keratoplasty versus repeat penetrating keratoplasty after failed penetrating keratoplasty: A systematic review and meta-analysis
Source: PLoS One. 2017 Jul 3;12(7):e0180468. doi: 10.1371/journal.pone.0180468 (PMC5495398; doi:10.1371/journal.pone.0180468)
Supplement: S1 File — (DOC) [file pone.0180468.s006.doc]

| **Section/topic** | **#** | **Checklist item** | **Reported on page #** |
| --- | --- | --- | --- |
| **TITLE** | | |  |
| Title | 1 | Endothelial Keratoplasty versus Repeat Penetrating Keratoplasty after Failed Penetrating Keratoplasty: A Systematic Review and Meta-Analysis | 1 |
| **ABSTRACT** | | |  |
| Structured summary | 2 | Objectives; methods; results; conclusions. | 2 |
| **INTRODUCTION** | | |  |
| Rationale | 3 | Introduction | 3-4 |
| Objectives | 4 | Introduction | 4 |
| **METHODS** | | |  |
| Protocol and registration | 5 | NA |  |
| Eligibility criteria | 6 | Materials and methods: Selection criteria | 5 |
| Information sources | 7 | Materials and methods: Literature Search Strategy | 4-5 |
| Search | 8 | Materials and methods: Literature Search Strategy | 4-5 |
| Study selection | 9 | Materials and methods: Selection criteria | 5 |
| Data collection process | 10 | Materials and methods: Data extraction and quality assessment | 6 |
| Data items | 11 | Materials and methods: Data extraction and quality assessment | 6 |
| Risk of bias in individual studies | 12 | NA |  |
| Summary measures | 13 | Materials and methods: Statistical analysis | 6-7 |
| Synthesis of results | 14 | Materials and methods: Statistical analysis | 6-7 |

Page 1 of 2

| **Section/topic** | **#** | **Checklist item** | **Reported on page #** |
| --- | --- | --- | --- |
| Risk of bias across studies | 15 | Materials and methods: Statistical analysis | 6-7 |
| Additional analyses | 16 | Materials and methods: Statistical analysis | 6-7 |
| **RESULTS** | | |  |
| Study selection | 17 | Results: Literature search | 7 |
| Study characteristics | 18 | Results: Study characteristics | 8 |
| Risk of bias within studies | 19 | NA |  |
| Results of individual studies | 20 | Results: Study characteristics | 8 |
| Synthesis of results | 21 | Results: Graft survival, Graft rejection, Visual acuity | 8-11 |
| Risk of bias across studies | 22 | NA |  |
| Additional analysis | 23 | NA |  |
| **DISCUSSION** | | |  |
| Summary of evidence | 24 | Discussions | 11-13 |
| Limitations | 25 | Discussions | 12-13 |
| Conclusions | 26 | Conclusion | 13-14 |
| **FUNDING** | | |  |
| Funding | 27 | Acknowledgments | 15 |

*From:*  Moher D, Liberati A, Tetzlaff J, Altman DG, The PRISMA Group (2009). Preferred Reporting Items for Systematic Reviews and Meta-Analyses: The PRISMA Statement. PLoS Med 6(7): e1000097. doi:10.1371/journal.pmed1000097

For more information, visit: **www.prisma-statement.org**.

Page 2 of 2
